# Supplementary material for: Novel Driver Strength Index highlights important cancer genes in TCGA PanCanAtlas patients
Source: PeerJ. 2022 Aug 11;10:e13860. doi: 10.7717/peerj.13860 (PMC9375969; doi:10.7717/peerj.13860)
Supplement: Supplemental Information 2 [file peerj-10-13860-s002.zip › COHORTS/genes plots/cohorts/2021_8_16_14_9_distribution_cohorts_SKCM.pdf]

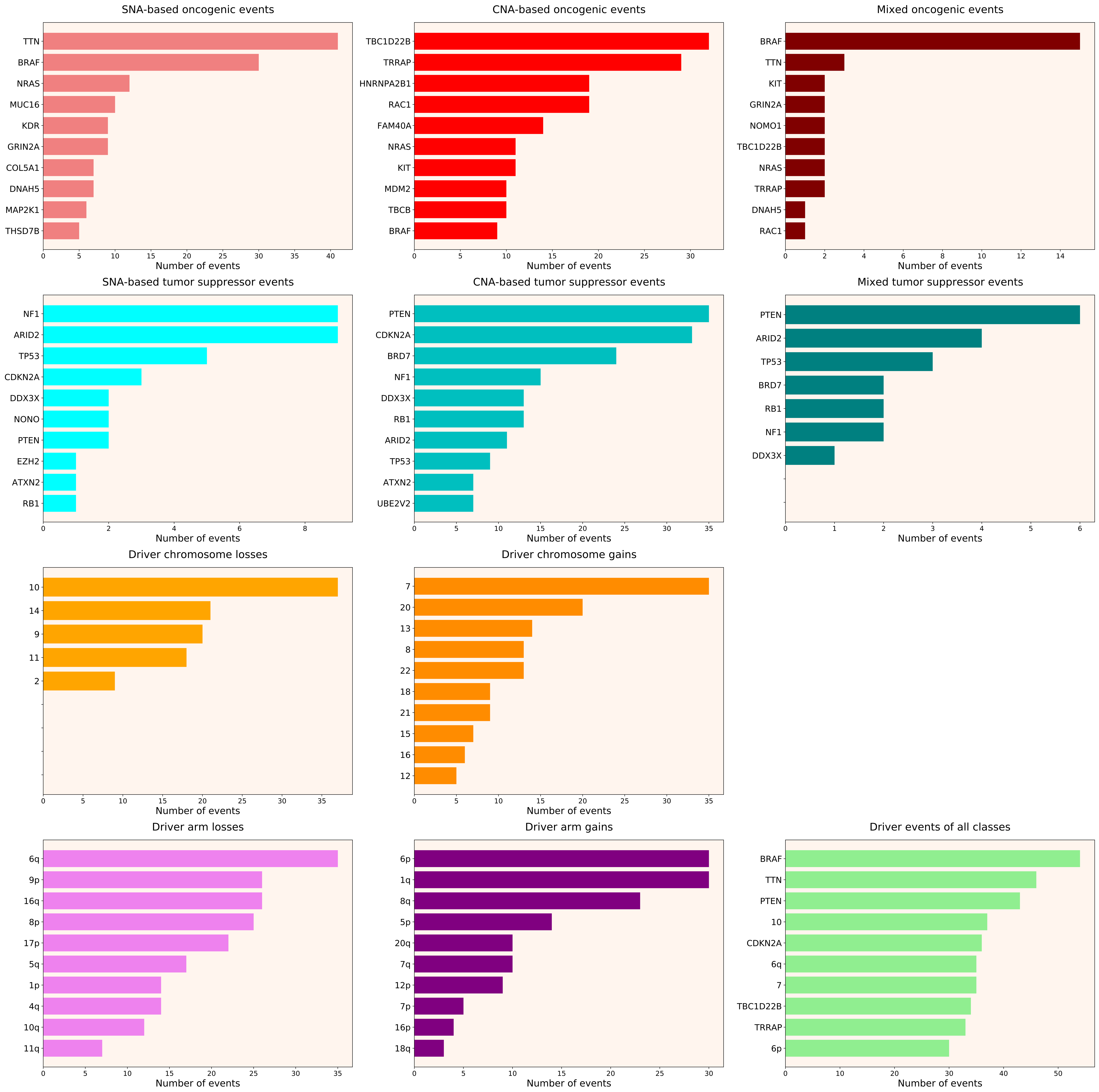
SNA-based oncogenic events
TTN
BRAF
NRAS
MUC16
KDR
GRIN2A
COL5A1
DNAH5
MAP2K1
THSD7B
Number of events
CNA-based oncogenic events
TBC1D22B
TRRAP
HNRNPA2B1
RAC1
FAM40A
NRAS
KIT
MDM2
TBCB
BRAF
Number of events
Mixed oncogenic events
BRAF
TTN
KIT
GRIN2A
NOMO1
TBC1D22B
NRAS
TRRAP
DNAH5
RAC1
Number of events
SNA-based tumor suppressor events
NF1
ARID2
TP53
CDKN2A
DDX3X
NONO
PTEN
EZH2
ATXN2
RB1
Number of events
CNA-based tumor suppressor events
PTEN
CDKN2A
BRD7
NF1
DDX3X
RB1
ARID2
TP53
ATXN2
UBE2V2
Number of events
Mixed tumor suppressor events
PTEN
ARID2
TP53
BRD7
RB1
NF1
DDX3X
Number of events
Driver chromosome losses
10
14
9
11
2
Number of events
Driver chromosome gains
7
20
13
8
22
18
21
15
16
12
Number of events
Driver arm losses
6q
9p
16q
8p
17p
5q
1p
4q
10q
11q
Number of events
Driver arm gains
6p
1q
8q
5p
20q
7q
12p
7p
16p
18q
Number of events
Driver events of all classes
BRAF
TTN
PTEN
10
CDKN2A
6q
7
TBC1D22B
TRRAP
6p
Number of events
